# Costs and quality of life of small-incision open cholecystectomy and laparoscopic cholecystectomy - an expertise-based randomised controlled trial

**DOI:** 10.1186/s12876-017-0601-1

**Published:** 2017-04-08

**Authors:** Mats H. Rosenmüller, Erik Nilsson, Fredrik Lindberg, Sten-Olof Åberg, Markku M. Haapamäki

**Affiliations:** Department of Surgery, Umeå University, SE-901 85 Umeå, Sweden

**Keywords:** Minilaparotomy, Health care costs, Surgical procedures

## Abstract

**Background:**

Health care providers need solid evidence based data on cost differences between alternative surgical procedures for common surgical disorders. We aimed to compare small-incision open cholecystectomy (SIOC) and laparoscopic cholecystectomy (LC) concerning costs and health-related quality of life using data from an expertise-based randomised controlled trial.

**Methods:**

Patients scheduled for cholecystectomy were assigned to undergo LC or SIOC performed by surgeons in two different expert groups. Total costs were calculated in USD. Reusable instruments were assumed for the cost analysis. Quality of life was measured using the EuroQol 5-D 3-L (EQ 5-D-3L), at five postoperative time points and calculated to Area Under Curve (AUC) for 1 year postoperatively. Two hospitals participated in the trial, which included both emergency and elective surgery.

**Results:**

Of 477 patients that underwent a cholecystectomy during the study period, 355 (74.9%) were randomised and 323 analysed, 172 LC and 151 SIOC patients. Both direct and total costs were less for SIOC than for LC patients. The total costs were 5429 (4293–6932) USD for LC and 4636 (3905–5746) USD for SIOC, *P* = 0.001. The quality of life index did not differ between the LC and SIOC groups at any time. Median values (25th and 75th percentiles (p25-p75)) for AUC at 1 year were as follows: 349 (337–351) for LC and 349 (338–350) for SIOC.

**Conclusions:**

In this expertise-based randomised controlled trial LC was a more costly procedure and quality of life did not differ after SIOC and LC. (ClinicalTrials.gov Identifier: NCT00370344, August 30, 2006).

**Electronic supplementary material:**

The online version of this article (doi:10.1186/s12876-017-0601-1) contains supplementary material, which is available to authorized users.

## Background

Over the past three decades, there has been substantial development in cholecystectomy techniques, as well as in the pre- and post-operative management of patients. The open surgical procedure with a large incision (OC) [1] has been succeeded by small incision open cholecystectomy (SIOC) [2, 3], laparoscopic cholecystectomy [4], single port laparoscopic cholecystectomy technique [5, 6], robotic cholecystectomy [7], and single port robotic cholecystectomy [8]. It is essential to scrutinise such changes in surgical technology in terms of cost and quality of life, to ensure fair distribution of resources. A meta-analysis of earlier randomised controlled trials showed no statistically significant differences, in terms of postoperative clinical outcomes, between SIOC and LC, but a significantly shorter operating time for SIOC [9]. Similar findings were reported in an expertise-based randomised controlled trial [10]. The aim of the present study is to compare LC and SIOC concerning costs and quality of life assessed by the EuroQol-5D-3L [11] using data from this expertise-based randomised controlled trial [12].

## Methods

Details concerning surgical teams and surgical techniques including intra-operative cholangiogram have been described earlier [10]. Primary outcomes were costs and health related quality of life, reported in the current paper, and pain. Secondary outcomes were complications within 30 days, operative time, length of hospital stay, conversion rate, frequency of ambulatory surgery and readmissions within 30 days, which together with pain have been reported earlier [10]. In short, SIOC was performed via a transverse incision over the right rectus muscle and laparoscopic cholecystectomy with a four-trocar technique. If the SIOC incision exceeded 8 cm, the operation was classified as an open cholecystectomy as this was the cut-off in the Swedish registry for gallstone surgery (GallRiks) [13]. Two hospitals participated in the trial, Umeå University Hospital and Lycksele County Hospital. The study was designed as a randomised pragmatic expertise-based trial [12, 14], where participating surgeons were asked to join one of two teams, performing either SIOC or LC, according to their personal preferences. Team members performed their specific method (SIOC or LC) as a first-choice operation, but converted to OC when necessary, and even began the operation using OC when neither LC nor SIOC were considered possible.

### Patients

Eligible patients were asked to enrol in the trial at the time when cholecystectomy was decided. Written informed consent was obtained before randomisation. The trial was not blinded.

### Inclusion and exclusion criteria

Patients aged 18 years or older with biliary disease where cholecystectomy was considered the best treatment according to published recommendations [15, 16] were asked to participate in the trial. Both elective and emergency patients were enrolled. Patients with acute cholecystitis, choledocholithiasis, jaundice, pancreatitis, obesity and co-morbidity were included if cholecystectomy was considered the best treatment. The exclusion criteria were: inability to understand given information, surgeons from either the LC team or the SIOC team unavailable for emergency cholecystectomy, cholecystectomy performed for malignant disease or suspicion of malignancy, and cholecystectomy performed as part of another operation.

### Randomisation

Randomisation was made using an internet-based system with computer-generated random numbers. Patients were automatically stratified at three levels: hospital, age (< or > = 70 years), and degree of priority (elective, emergent, or surgery for life-threatening disease). The patients were randomised when the decision for surgery was made.

### Economic analysis

The economic analysis was conducted from a societal perspective, calculating both direct public healthcare costs and indirect costs generated by the loss of productivity (sick leave). Cost generating posts in health care were calculated from the time the patient was admitted for surgery. Preoperative investigations (preoperative radiology, preoperative visits to outpatient clinic, etc.) were not included. Costs for pre-, intra-, and postoperative endoscopic retrograde cholangiogram (ERC), sphincterotomy and endoscopic stone extraction were regarded as a part of the operative procedure and were thus included. Detailed calculations of cost items were performed in one hospital (Umeå University Hospital). All costs are given as USD. Where reliable item costs could be found, they were retrieved from the Hospital Department of Economics (costs for perioperative and secondary cholangiogram, in hospital stay, postoperative recovery unit). Other costs were calculated manually, (disposables, reusable instruments, including laparoscopic equipment). Costs for LC were calculated with the presumption of reusable trocars. Personnel costs per minute were calculated using mean wages for each category of personnel obtained from the Department of Economics, Umeå University Hospital. Costs for social benefits and employer fees, at a rate of 43.71% (mean for hospital staff), were added to the mean wages. Operational costs were calculated with one resident and one senior surgeon participating from incision to last stich for each operation. A standard cost for counselling and administration (hospital record, sick leave certificate and prescription of drugs) were added. The cost for surgical theatre staff was calculated from the moment patients arrived at the theatre until they left for the recovery unit, individually for each patient. All patients were estimated to spend 4 h in the postoperative recovery unit. Patients undergoing ambulatory surgery were estimated to spend 8 h in the surgical ward. An average cost for ERC, sphincterotomy and endoscopic stone extraction of 1216 USD, was estimated by the Hospital Department of Economics. Indirect costs due to loss of production were calculated based on mean wages obtained from Statistics Sweden (SCB) for men and women, respectively, including social benefits and employer fees at a rate of 41.15% (mean for all employed). Indirect costs were calculated only for patients that reported taking a sick leave from work due to the operation. Cost-generating posts are listed in Table 1.

**Table 1:** Cost-generating items and unit costs

*Cost item*	*Unit cost, USD*	*Cost category*
Preoperative ERC	1216.00	Other HCC
Perioperative cholangiogram	295.79	Op cost
Material costs/operation		Op cost
*LC*	481.38	
*LC converted to OC*	559.51	
*SIOC*	419.67	
*SIOC converted to OC*	419.67	
Surgeons costs/min	4.27	Op cost
Anaesthesia cost/min	2.64	Op cost
Other staff costs/min	3.66	Op cost
Facilities costs/min	1.60	Op cost
Fixed costs operating staff/operation	757.11	Op cost
Recovery unit costs/operation	267.37	Other HCC
Secondary cholangiogram costs	197.14	Other HCC
Postoperative ERC cost	1216.00	Other HCC
Hospitalisation cost/day	817.30	Other HCC
Readmission cost/day	817.30	Other HCC
Sick leave costs men/day	210.67	Indirect costs
Sick leave costs women/day	181.18	Indirect costs

*Other HCC* other health care costs, *Op cost* cost generated by operation, *LC* laparoscopic cholecystectomy, *OC* open cholecystectomy, *SIOC* small-incision open cholecystectomy

### Health-related quality of life

Health-related quality of life was assessed using the EQ 5-D-3L (EuroQol Group, Rotterdam, The Netherlands [11]) which consists of five questions concerning patient mobility, self-care, usual activities, pain/discomfort and anxiety/depression. In this study, a value set derived from the Swedish population was used [17]. The EQ-5D-3L index values were further calculated to AUC and quality adjusted life years (QALYs), please see *Sample size, analysis and statistics.*

### Data collection

The operating surgeon registered the operative data (except for operative time) in the Swedish registry for gallstone surgery, GallRiks [13]. An independent assessor, as defined by GallRiks, registered operative time, length of hospital stay and complications within 30 days. Complications were graded according to Dindo-Clavien [18]. Health-related QoL was recorded by the patient before surgery and on postoperative days 3, 7, 11, and 30, as well as 1 year after surgery. The patient reported length of sick leave at 30 days. Data from GallRiks were controlled against patient hospital records for all patients. Where registration errors were detected, they were corrected.

### Sample size, analysis and statistics

The power calculation to determine the sample size for the trial was based on earlier publications on cholecystectomies where the main differentiating factor between the two interventions was duration of the operation. It was assumed that SIOC would take 16 min less compared to LC, based on three previous studies [19–21]. The calculation was made for 90% power, 5% significance level and an anticipated data loss of 25%. With these assumptions, it was calculated that the study should consist of at least 350 patients. The sample size was estimated to be sufficient for detecting significant differences of routine costs, but not for comparing relatively rare complications between groups. All analyses were made with the intention-to-treat principle. Quantitative results were presented as median values, 25 and 75^th^ percentiles whenever the distribution of the data was skewed. A non-parametric Mann-Whitney U test was used for statistical tests of significance. AUC (Area Under Curve) values up to 1 year were calculated using the EQ-5D-3L scores at five different postoperative time points and the assumption that the change between time points was linear. Missing EQ-5D-3L values were imputated if the case had at least two registered genuine EQ-5D-3L values at different time points, otherwise the case was excluded from AUC calculation. We used the principle to add (or subtract) the mean change between specific time points for the group (LC or SIOC) to the last genuine value to generate the value for the next time point if it was missing. In all, 128 imputations for missing values were added to the 1372 genuine values. Thirty-three cases were excluded because of more than three missing EQ-5D-3L scores. The EQ-5D-3L scores and AUC values were calculated from raw data using Microsoft® Excel for Mac version 14.2.3 (Microsoft Corporation, Redmond, Washington, USA), and Stata® software release 13.1 (StataCorp LP, College Station, Texas, USA) was used for statistical calculations.

## Results

**Fig. 1:**
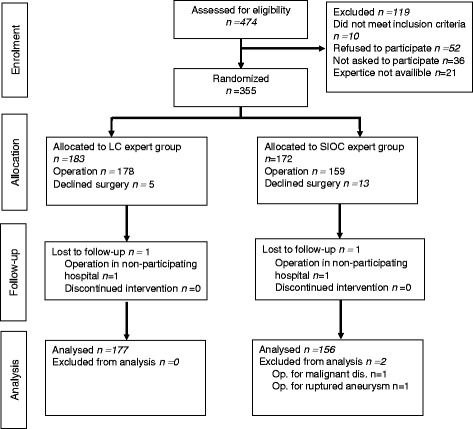
CONSORT diagram for the trial. Analysis according to the ‘intention to treat’ principle. (Modified reprint from *Br J Surg* 2013;100(7): 886–894, with written permission from original publisher John Wiley and Sons)

A flow diagram describing the progress from enrolment to analysis according to the CONSORT statement [22] is presented in Fig. 1. Of 477 assessed patients, 355 (74.9%) were randomised. Twenty-two patients (LC 6 and SIOC 16) were excluded from analysis of which 18 declined surgery, two were operated on at a non-participating hospital and 2 were operated on for other non-related disease. Finally 333 were analysed, 177 LC and 156 SIOC patients. Of these 333 patients, postoperative QoL data was available and analysed for 290, 156 LC and 134 SIOC patients. Study protocol violations were noted for 33 patients. In accordance with the CONSORT Statement [22] cases with protocol violations were not excluded, but were analysed according to the original randomisation.

### Baseline data

Age, sex, preoperative EQ-5D-3L score, proportion of smokers, proportion employed and education level were similar between the groups (Table 2). The SIOC-group had a longer time between decision for surgery and operation [10], 156 versus 103 days, respectively (*P* = 0.03), slightly more emergency admissions, and more cases with acute cholecystitis than the LC group, 25.6 and 14.7%, respectively (*p* = 0.012).

**Table 2:** Patient characteristics for 333 patients

	LC	SIOC
	*n* = 177	*n* = 156
Male/Female % (n)	38/62 (68/109)	39/61 (61/95)
Age in years, median (range)	52 (18–84)	51.5 (20–84)
Preoperative EQ-5D-3L score, Median (p25, p75)	0.9349 (0.8337–0.9349)	0.9349 (0.8337–0.9694)
Patients with QoL data	153/177	131/156
Employed % (n)	58 (89/154)	55 (73/133)
Smoker % (n)	16 (23/148)	10 (13/132)
Higher education % (n)	29 (44/150)	32 (43/135)

*LC* laparoscopic cholecystectomy, *SIOC* small-incision open cholecystectomy, *EQ-5D-3L* EuroQol-5D-3L instrument for assessment of quality of life, *QoL* quality of life

Proportion of subgroup is presented when there was missing data

### Outcomes

The median value for direct cost was (p25-p75) 4210 USD (3851–5073) for the LC group and 3963 USD (3512–4848) for the SIOC group, *P* = 0.002. Operation costs and total costs, including indirect costs, were significantly higher for the LC group, *p* = <0.001 and *p* = 0.003 respectively (Table 3).

**Table 3:** Cholecystectomy costs for all patients

	LC (*n* = 177)	SIOC (*n* = 156)	P ^b^
Total costs with loss of production (sick leave) included ^a^			
Median	5442.06	4783.90	0.003
p25-p75	4323–7000	3919–5906	
Total costs, loss of production omitted ^a^			
Median	4210	3963	0.002
p25-p75	3851–5073	3512–4848	
Operation costs^a^			
Median	3183	2882	<0.001
p25-p75	2959–3479	2638–3320	

^a^All costs are given in USD

^b^Comparision with Mann-Whitney test

*p25-p75* 25^th^ and 75^th^ percentiles

**Fig. 2:**
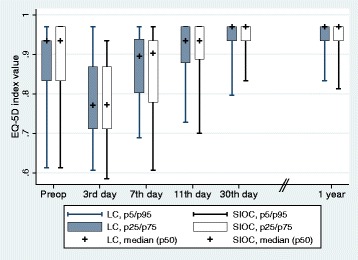
Health-related quality of life (QoL) scores before operation and at five postoperative time points, measured with the EQ-5D-3L scale, with scores ranging from 0 to 1, where 1 is the best possible health-related quality of life. Values are presented as the median and as percentiles 25 to 75^th^ (*box*) and 5 to 95^th^ percentile range (whisker)

EQ-5D-3L-AUC within 1 year was, median, (p25-p75) 349 (337–351) for the LC group and 349 (338–350) for the SIOC group, *P* = 0.8096. QALYs had a median of 0.9639 (0.9313–0.9683) and 0.9636 (0.9343–0.9679) in the LC group and SIOC group, respectively. EQ-5D-3L-AUC at 30 days was, median (p25-p75) 25.17 (23.70–25.85) for the LC group and 25.10 (23.67–25.76) for the SIOC group. Index values for EQ-5D-3L at all time points are shown in Fig. 2. Subgroup analyses for costs and QoL of patients that had emergency cholecystectomy, elective cholecystectomy, complications and no complications are presented in Additional file 1: Table S1.

## Discussion

In this expertise - based RCT, LC carried significantly higher costs than SIOC, direct as well as indirect costs but there were no significant differences in health-related QoL between the LC and SIOC group when measured with EQ-5D-3L.

In accordance with previous studies [9], the present trial has not demonstrated significant differences in complication rate between LC and SIOC [10]. It was designed as a pragmatic randomised expertise-based trial [14] with the intention to minimise exclusions and differential bias [12, 14]. The expertise-based setting made it possible to include patients otherwise unfit to be included in a regular trial such as patients with prior upper gastro-intestinal surgery, patients with mild biliary pancreatitis, and patients with acute cholecystitis. Therefore, we had a high percentage of patients assessed for cholecystectomy included in the trial (75%). Operating times in the present study were longer for LC than for SIOC in accordance with previous studies [9]. Our operating times were long reflecting the high inclusion ratio with the ambition to include also acute operations. A setting with disposable trocars and clip applier would have added an estimated 304 USD to the perioperative costs for the LC group.

There are some potential limitations to this study. First, after completion of the study we found that time from randomisation to cholecystectomy was longer for SIOC than for LC patients [10]. We had fewer SIOC surgeons than LC surgeons, 6 vs 11. This increased the time on waiting list for SIOC patients, some of which had to be operated emergently. Consequently, more patients with acute cholecystitis appeared in the SIOC group, which explains the slightly higher pain score and VAS-QoL score preoperatively for SIOC patients [10]. However, in the present report only the validated EQ-5D-3L instrument was utilised for quality of life estimation. Second, measuring indirect costs (e.g. sick leave) can be considered controversial, as it may reflect local traditions as well as political and cultural differences. In Sweden, a doctor’s certificate is required after the seventh’ day of sick leave. The study protocol stated that sick leave certificates should not routinely be written after uncomplicated surgery. In this study, LC patients had a higher cost for loss of production (indirect costs) without QoL difference. This might reflect that surgeons in the SIOC group, after the SIOC training phase were more compliant to the study protocol than the LC group surgeons. This imbalance in sick leave was also found in an earlier study [23].

Several reasons necessitate a discussion of cost-effectiveness in treatment of gallstone disease. The increasing population age in industrialized countries escalates health care costs [24]. Sphincterotomy has separated treatment of bile duct stones and gallbladder stones [25, 26], although SIOC, with choledochotomy, and primary closure of the common bile duct during cholecystectomy is safe, effective and inexpensive when performed by trained surgeons [27]. The decline in training in open surgery for residents [28] is of concern for the treatment of gallstone disease [29, 30]. After appropriate training, SIOC is an alternative to LC wherever the health-care budget is limited, not only in the third world [31].

## Conclusions

From this trial we conclude that LC is more costly, but SIOC and LC are comparable in terms of QoL.

## Additional file

Additional file 1: Table S1. Subgroup analyses. Additional file holds a supplementary table (Table S1) with subgroup cost and QoL analysis of patients that had a) emergency cholecystectomy, b) elective cholecystectomy, c) complications and d) no complications. (DOCX 18 kb)

## Abbreviations

AUC: Area under curve
EQ 5-D-3L: EuroQol 5-D 3-L
ERC: Endoscopic retrograde cholangiogram
GallRiks: Swedish registry for gallstone surgery
LC: Laparoscopic cholecystectomy
OC: Open cholecystectomy
QALYs: Quality adjusted life years
SCB: Statistics Sweden
SIOC: Small-incision open cholecystectomy
USD: United States Dollar
