# Supplementary material for: Costs and quality of life of small-incision open cholecystectomy and laparoscopic cholecystectomy - an expertise-based randomised controlled trial
Source: BMC Gastroenterol. 2017 Apr 8;17:48. doi: 10.1186/s12876-017-0601-1 (PMC5385047; doi:10.1186/s12876-017-0601-1)
Supplement: Additional file 1: Table S1. — Subgroup analyses. Additional file holds a supplementary table (Table S1) with subgroup cost and QoL analysis of patients that had a) emergency cholecystectomy, b) elective cholecystectomy, c) complications and d) no complications. (DOCX 18 kb) [file 12876_2017_601_MOESM1_ESM.docx]

### Additional file 1

Table S1. Subgroup analyses

Cholecystectomy costs and 1-year EQ 5D-3L AUC for emergency cholecystectomy, elective cholecystectomy, patients with complications and patients without complications.

|  | LC | SIOC | P^2^ |
| --- | --- | --- | --- |
| *Emergency cholecystectomy*  Total costs, loss of production omitted^1^ |  |  |  |
| Median | 5110 | 4591 | 0.092 |
| p25-p75 | 4298-6658 | 4080-5581 |  |
| N= | 52/177 | 55/156 |  |
|  |  |  |  |
| 1-year EQ 5D-3L AUC |  |  |  |
| Median | 349.26 | 349.09 | 0.388 |
| p25-p75 | 331.25-350.51 | 338.28-350.28 |  |
| N= | 47/177 | 52/156 |  |
|  |  |  |  |
| *Elective cholecystectomy* |  |  |  |
| Total costs, loss of production omitted^1^ |  |  |  |
| Median | 4021 | 3728 | <0.001 |
| p25-p75 | 3720-4460 | 3367-4157 |  |
| N= | 125/177 | 101/156 |  |
|  |  |  |  |
| 1-year EQ 5D-3L AUC |  |  |  |
| Median | 348.55 | 346.44 | 0.710 |
| p25-p75  N= | 335.49-350.44  114/177 | 334.53-350.38  87/156 |  |
|  |  |  |  |
| *Patients with complications*  Total costs, loss of production omitted^1^ |  |  |  |
| Median | 7124.5 | 12519.6 | 0.247 |
| p25-p75 | 4725-11052 | 4951-15576 |  |
| N= | 19/177 | 17/156 |  |
|  |  |  |  |
| 1-year EQ 5D-3L AUC |  |  |  |
| Median | 334.18 | 322.43 | 0.212 |
| p25-p75 | 297.02-350.58 | 273.92-347.19 |  |
| N= | 19/177 | 15/156 |  |
|  |  |  |  |
|  |  |  |  |
|  |  |  |  |
| *Patients without complications* |  |  |  |
| Total costs, loss of production omitted^1^ |  |  |  |
| Median | 4116 | 3821 | <0.001 |
| p25-p75  N= | 3824-4845  158/177 | 3478-4528  139/156 |  |
|  |  |  |  |
| 1-year EQ 5D-3L AUC |  |  |  |
| Median | 348.98 | 349.09 | 0.834 |
| p25-p75 | 338.51-350.51 | 338.39-350.50 |  |
| N= | 142/177 | 124/156 |  |
|  |  |  |  |

LC=laparoscopic cholecystectomy, SIOC= small-incision open cholecystectomy, EQ-5D-3L= EuroQol-5D-3L instrument for assessment of quality of life, AUC= Area Under Curve, Loss of production=costs of sick leave, p25-p75= 25^th^ and 75^th^ percentiles

^1^All costs are given in USD

^2^Comparision with Mann-Whitney test.
